# Human immunodeficiency virus infection disclosure status to infected school aged children and associated factors in bale zone, Southeast Ethiopia: cross sectional study

**DOI:** 10.1186/s12887-018-1336-z

**Published:** 2018-11-15

**Authors:** Bikila Lencha, Gemehu Ameya, Zanebe Minda, Feyissa Lamessa, Jiregna Darega

**Affiliations:** 1 Department of Public Health, Goba Referral Hospital, Maddawalabu University, Bale-Goba, Addis Ababa, Ethiopia; 2 Department of Medical Laboratory Science, College of Medicine and Health Sciences, Arba Minch University, P.O. Box: 21, Arba Minch, Ethiopia; 3 Department of Nursing, Goba Referral Hospital, Maddawalabu University, Bale-Goba, Addis Ababa, Ethiopia; 4 Department of Nursing, College of Medicine and Health sciences, Ambo University, Ambo, Ethiopia

**Keywords:** Caregivers, Health care workers, HIV-positive status disclosure, School aged children

## Abstract

**Background:**

Human immunodeficiency virus (HIV) positive status disclosure is an essential component of Pediatric care and long term disease management. Children have a right to know their HIV diagnosis result. However, Pediatric HIV disclosure is complex and varies in different communities. This study aimed to assess the prevalence of HIV-positive status disclosure to infected children and associated factors among caregivers of infected children.

**Methodology:**

A facility based mixed methods research design study was conducted in Bale Zone of South East Ethiopia. Randomly selected caregivers of HIV-positive children were interviewed using structured questionnaires for quantitative study and 17 in-depth interviews of health care workers and caregivers were conducted for qualitative data. Content analysis was done for qualitative data and logistic regression analysis was used to see the association between different variables and HIV-positive disclosure status. Odds ratio with 95% CI was computed to determine the presence and strength of the associated factors.

**Results:**

A total of 200 caregivers of school aged (6–14 years) children participated in the study. Only 57 (28.5%) of the care givers disclosed HIV-positive status to the child for whom they were caring. The main reason for disclosure delay was due to fear of negative consequences, perception on maturity of the child, and fear of social rejection and stigma. Having social support [AOR = 2.7, 95% CI: (1.1–6.4)], caring for a child between 10 and 14 years with HIV [AOR = 6.5, 95% CI: (2.1–20.2)], a child diagnosed with HIV at age > 5 years [AOR = 2.8, 95% CI: (1.1–7.1)], and children on antiretroviral therapy (ART) with follow-up for > 5 years [AOR = 4.7, 95% CI: (1.8–11.2)] had significant association with HIV- positive status disclosure to infected children.

**Conclusion:**

The frequency of HIV infection disclosure to infected children was very low in our cohort. Having social support, having an older child with HIV, a long period of ART follow-up and HIV diagnosis after age of five years were positively associated with HIV-positive status disclosure to infected children. Giving age appropriate counselling to children, social support to the caregivers and working on related factors are very important to improve the observed low disclosure status.

**Electronic supplementary material:**

The online version of this article (10.1186/s12887-018-1336-z) contains supplementary material, which is available to authorized users.

## Background

Globally, Pediatric human immunodeficiency virus (HIV) infection continues to be a major problem. A 2013 WHO report showed that approximately 3.3 million children younger than 15 years are living with HIV, with about 88% of them living in sub-Saharan Africa [1]. The annual number of new infections among children was almost halved since 2010. Regardless of this significant reduction, the number of children newly infected with HIV remains unacceptably high. According to 2017 global HIV statistics, there are 1.8 million children living with HIV. Among these about 10% of them were new infections. Of newly infected Pediatric cases, more than half of them were from eastern and southern Africa. The majority of pediatric HIV was acquired from their infected mothers vertically or during breast feeding [2]. The pediatric HIV-positive population in Ethiopia is mostly an older age group that probably vertically transmitted in earlier years when mother to child HIV transmission prevention coverage was not well established [3]. The current expansion of ART plays a significant role in reducing mortality of infected children. Studies revealed that many HIV-positive children on ART do not know their HIV status [4, 5].

The American Academy of Pediatrics strongly encourages disclosure of HIV-positive status to school-age children [6]. It is believed that disclosure of HIV status to infected children has tremendous benefit in improving the treatment outcome. The disclosure resulted in mental relief for caregivers from the burden of keeping a secret [7]. It is belived that children have the right to know their HIV status. Further more, disclosure of HIV-positive status following the child’s diagnosis is very important to ensuring child wellbeing. Different factors may be associated with lack of disclosure of HIV status to infected children. The depth of HIV status information to be shared with children, the manner and time of disclosure are things to be considered by caregivers and healthcare workers [8].

Early disclosure is more appropriate than immediate and unplanned disclosure upon entrance into the adult clinic, and also helps to reduce HIV transmission [9]. The prevalence of pediatric HIV disclosure to infected children varies widely throughout the world. In developed countries such as the United States, HIV diagnosis disclosure was reported to be up to 100% according to a study conducted in 2009 [10]. In developing countries, caregivers often do not disclose HIV-positive status to infected children. Vreeman et al. showed that the disclosure of HIV-positive status was as low as 1.6% in Kenya [11]. HIV disclosure practices in sub-Saharan African countries remain complex due to the immense influence of politics, culture and HIV surveillance limitation [12]. Studies of HIV disclosure in children are very limited in Ethiopia, and available studies are more prevalent in Addis Ababa and the Northern part of the country. In Ethiopia, studies showed that the prevalence of disclosure among caregivers’ of children varies from 16.3 to 39.5% [13–15]. In addition to the low frequency of disclosure in Ethiopia, little is known about the associated factors of HIV disclosure in school-aged children.

In previous studies which were conducted in other areas, factors like a child’s age, the age of diagnosis, being on antiretroviral therapy (ART), caregiver-reported child depression symptoms, caregivers relation with the child and loss of a family member were found to be associated with HIV infection disclosure. Children with a deceased father tended to be more likely to know their status than non-orphans [16, 17]. These factors may vary depending on factors like sociodemographic characteristics, sociocultural aspects, awareness of care givers and knowledge of health professionals. Therefore, it is important to know the frequency of disclosure, its associated factors, the opinion and experience of caregivers and health care workers (HCWs) to design an appropriate intervention suitable with specific living and the cultural context of the society. Thus, this study aimed to assess the prevalence of HIV-positive status disclosure and associated factors among caregivers of HIV-infected children in Bale Zone, South East Ethiopia.

## Methods

### Study setting and period

The study was conducted in Bale Zone of south east Ethiopia at a distance of 430 km from Addis Abeba. The zone has four hospitals (Goba, Ginnir, Robe and Dallomana) and five health centers (Gasara, Goro, Agarfa, Dinsho and Barbare) that were giving pediatric ART service during the data collection period. Bale zone was selected as no studies on pediatric HIV-positive disclosure have been conducted there. There are also a large number of children followed in the ART clinic in the governmental health facility in the zone.

### Study design and population

An institutional based mixed methods research design was conducted. Both qualitative and quantitative methods were used to collect the data. The caregivers of children aged between 6 and 14 years who were on ART were included from selected pediatric ART clinics for quantitative study. The caregivers were randomly selected for quantitative study. For those children who came by themselves, their caregivers were contacted with the help of pediatric ART service providers. Health care workers (HCWs) working in the pediatric ART clinics and caregivers were purposively included in the study for the in-depth interviews. The health care workers working in ART clinics during data collection and caregivers coming with their children were selected for in-depth interviews.

### Sample size determination and sampling methods

The sample size was calculated using a single population proportion formula with estimated proportion of disclosure among school-aged children to be 50% due to a lack of previous studies conducted in the same areas. Marginal error was assumed to be 5 and 95% confidence interval. The estimated sample size was 384 subjects, and a population correction formula was used because of the low number (< 10,000) of school-age children on ART in the study area. Finally, after adding 10% non-response rate, the sample size was estimated to be 201. Two hospitals and two health centers were randomly selected by lottery method from those institutions giving pediatric ART service. The total sample size was allocated proportionally to the total number of children on treatment in each of the selected ART clinics. The children were selected by simple random sampling technique using sample frames recorded on computer data after excluding drop-out, transfer-out and lost to follow-up. The children’s corresponding caregivers were interviewed in a separate room while they came for follow-up to the ART clinic. For the qualitative study, purposive sampling was used and 17 in-depth interviews were conducted.

### Data collection methods and instruments

Data were collected using the structured interviewer administered questionnaire which was adapted after reviewing related literature [14, 15, 18]. The questionnaire was first prepared in English (Additional file 1) and then translated into local languages (Amharic and Afaan Oromo) by language experts and back translated into English to check its consistency. Data were collected by 6 health professionals working in the pediatric ART clinics. Medical records of HIV-positive children were reviewed for date of HIV diagnosis, current WHO treatment stage and the most recent CD4 count.

For the in-depth interview, a semi-structured interview guide (Additional file 1) was developed from pertinent literature [7, 18] and held with caregivers and HCWs to explore and understand perceptions and experiences towards disclosure of HIV-positive status to infected children. The interview was facilitated by investigators using a guide and it was tape recorded. Notes were taken by one of the investigators. Seventeen in-depth interviews were conducted until thematic saturation was reached.

### Data quality control

Training was given for data collectors for two days on the objectives, contents, and procedures of the data collection. The questionnaire was pretested on 5% of the sample in non-selected hospitals before the actual data collection and revised prior to data collection. Data was checked for completeness during the data collection by supervisors and investigators. The realiablity of the questionnaire was checked using cronbach’s alpha and it was above 0.7. Qualitative data was transcribed on the same day after data collection and appropriate corrections were made for the next day.

### Data processing and analysis

Data were entered using Epi info version 7.0 and exported to Statistical Package for Social Sciences (SPSS) version 21 for analysis. Descriptive statistics were used to assess the socio-demographic characteristics of caregivers and children. HIV-positive status disclosure to infected children was dichotomized to ‘yes’ and ‘no’ based on caregivers’ self-report.

Bivariable and multivariable logistic regression analyses were used to determine factors associated with HIV infection disclosure status to children. Variables with a *p*-value of ≤0.25 in univariate logistic regression were included in a multiple logistic regression model to control for potential confounders. The stepwise backward variable selection method was used in the multivariable analysis. *P*-values < 0.05 were considered to declare statistical significance in the models.

For the qualitative data, the recorded data was transcribed verbatim and then translated into English word-for-word. The content analysis was used by sorting information, looking for similarities and differences, and developing appropriate codes. Then, similar codes were used to make categories. Finally, the qualitative data was summarized and direct quotations were used to present the data along with the quantitative findings.

## Results

### Socio-demographic characteristics of HIV-positive school aged Children’s caregivers

A total of 200 caregivers of children aged from 6 to 14 years participated in the study with a response rate of 99.5%. About two-thirds of the respondents were female and nearly half of the respondents were within the age group from 31 to 40 years. About 70% of the respondents were married and 27% of them were unemployed. A majority of the respondents (68%) were urban residents and nearly three-fourths of the caregivers were biological parents of the children for which they cared (Table 1).

**Table 1:** Socio-demographic characteristics of the HIV-positive school aged children’s caregivers of Bale Zone, Southeast Ethiopia

Variables	Categories	No.	%
Age (years)	19–30	52	26.0
	31–40	93	46.5
	>40	55	27. 5
Sex	Male	66	33.0
	Female	134	67.0
Religion	Orthodox	102	51.0
	Muslim	68	34.0
	Protestant	30	15.0
Marital status	Single	21	10.5
	Married	139	69.5
	Divorced	10	5.0
	Widowed	30	15.0
Occupation	Unemployed	54	27.0
	Government Employee	32	16.0
	Merchant	38	19.0
	Farmer	52	26.0
	Others^a^	24	12.0
Income (Birr)	≤500	60	30.0
	501–1000	78	39.0
	≥1000	62	31.0
Educational status	Unable to read and write	64	32.0
	Able to read and write	62	31.0
	Primary (1–8)	20	10.0
	Secondary (9–12)	40	20.0
	Tertiary (12 and above)	14	7.0
Residence	Urban	136	68.0
	Rural	64	32.0
Relation of caregiver to the child	Biological parents	143	71.5
	Non-biological parents	41	20.5
	Others^b^	16	8.0

*Others*^a^ students and pensioners, *Others*^b^ caregivers from the camp (foster parents)

### Socio-demographic characteristics of the HIV-positive school aged children

More than half of the HIV-positive children were boys. The mean age of the children was 9.9 years with ±2.6 years standard deviation (SD). About 55% of children were between 10 and 14 years. The mean age at which the children were diagnosed was 5.6 ± 2.4 years SD. All the children were on ART during the time of the caregivers’ interview, and the mean duration of stay on ART was 4.4 ± 2.4 years. The majority of the children (82%) were attending school. About 43% of the children on ART were taking the drug by their own initiative. According to caregivers report, more than half of the children were cared for by single parents (Table 2).

**Table 2:** Socio demographic characteristics of the HIV-positive school aged children in Bale Zone, Southeast Ethiopia

Variable	Categories	No.	%
Age of the child	6–9 years	91	45.5
	10–14 years	109	54.5
Sex of the child	Male	108	54.0
	Female	92	46.0
Age at diagnosis of HIV	1–5 years	113	56.5
	6–11 years	87	43.5
Age when ART was initiated	1–5 years	95	47.5
	6–11 years	105	52.5
Duration on ART	1–5 years	136	68.0
	6–12 years	64	32.0
Responsible to take ART	Yes	86	43.0
	No	114	57.0
Schooling	Yes	164	82
	No	36	18
With whom the child is currently living?	Biological parents	142	71.0
	Non-biological parents	46	23.0
	Foster parents (camp)	12	6.0
Did the child lose any of their biological parents?	Yes	104	52.0
	No	96	48.0

### Magnitude and reasons of disclosure of HIV-positive result to infected school aged children

Based on caregiver reports, only 28.5% of the children were disclosed their HIV-positive status. About 91% of the disclosed children were in the age range from 10 to 14 years while the remaining were from 6 to 9 years. The mean age at disclosure was 10.7 ± 1.8 years. More than half of the caregivers’ children in the age range between 10 and 14 years were not disclosed about their HIV-positive status.

The major reasons for disclosure were repeated questions from the children to know why they took the drugs, followed by positive perception on child maturation and ability of understanding the information. In-depth interview of the caregivers also supports this idea. The caregiver believed that the appropriate age for disclosure is 10 years or above. In qualitative study, the age of children and their ability to understand were important issues to consider regarding disclosure of HIV-positive status to infected children. Health care workers had almost similar opinions concerning the preferred age of disclosure. The majority of caregivers (70.5%) preferred the age group between 10 and 14 years to disclose HIV-positive status, whereas about 27% of them preferred children aged 15 years and above. However, a 32-year-old health worker with 2-years work experience in ART mentioned that a 14-year-old female who was not disclosed started a relationship with her boyfriend and infected him with the virus.

Of the 57 children to whom the HIV-positive result was disclosed, nearly two-thirds of them were disclosed by their biological parents followed by health worker (16%) and relatives (9%). In the in-depth interview, the HCWs said that willingness of the family is important to disclose the HIV-positive status to the infected children. In addition, HCWs said that the families have the responsibility to start the process of disclosure at home because they stay with the child for a long time. HCWs also said that their role is to support the caregiver’s disclosure, teach the process and help caregivers with the day to day challenges about disclosure.

### Reasons for non-disclosure of HIV-positive status

**Fig. 1:**
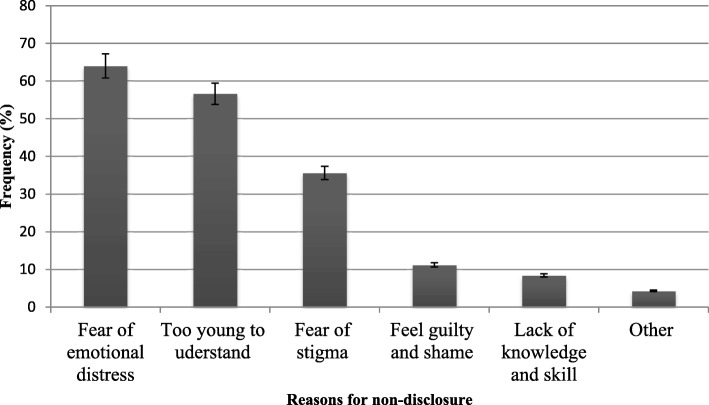
Caregivers’ reasons for non-disclosure of HIV infection to their children in Bale Zone, Southeast Ethiopia

Out of the total study participants, 143 (71.5%) of the caregivers did not disclose the diagnosis result of HIV infection to their children. Of these participants, 91 (64%) of the care-givers delayed disclosure for fear of negative consequences for the child (fear of emotional distress), 81 (56.6%) reported that the child was too young to understand the diagnosis, and 51 (35.6%) of them replied the child would be socially rejected (fear of stigma and discrimination) (Fig. 1).

In in-depth interviews, all the caregivers mentioned that the age of the children is the main factor hindering disclosure. The participants also mentioned that the child could not understand the information and may disclose to other people. And health care workers also shared their ideas. A 42 year old female HCW with 4-years experiences in ART clinic said that *“Families believe that they are the cases for themselves and feel guilty and ashamed. Fear of stigma and age of the child were also another problem. Family believe that the child couldn’t understand at this age and disclosure leads to emotional abuse.”*

Health care workers were asked for their experience about disclosure, and they responded that a sudden disclosure of infection status to HIV-infected children in the school and from families other than biological parents ended up with bad consequences. Children became emotional when they heard their diagnosis from the person they did not expect and in an unexpected situation. One of the HCWs said that “*Once upon a time the foster father of a child comes home after drinking alcohol and said ‘keep silent and take your long life medication’ to the child. Then the child went to the kitchen and took insecticide medication and died. Therefore, sudden disclosure has bad consequences”* (34-year-old HCW with 2 years expriance).

All the HCWs and caregivers were also asked about the way of disclosure. The participants responded that disclosure should be a process, not a one-time activity. They said ‘How much does the child understand about HIV/AIDS?’ is the main question that should be addressed. One of the HCWs said “*Those 10 years and above should be told about the diagnosis step by step. The child will understand you through time. It needs time. The HCW will stay with the child not more than 1hr. If the health worker discloses within this short time, the child will become emotional. Therefore, it should be at home by taking time and convincing the child slowly”* (42-year-old HCW with 4 years experience).

### Factors associated with disclosure of HIV-positive status to HIV-infected children

Factors associated with disclosure of HIV-positive status to infected children was assessed by binary logistic regression. In univariate analysis, gender of caregivers with whom the children were living, HIV status of care giver, respondent residence, presence of social support, age of the children, schooling status of the children, age at which the child was diagnosed with HIV and duration of ART had *p*-values less than 0.25 and the variables were selected for multivariate analysis. In multivariate analysis, presence of social support for caregiver, age of children, age at which children were diagnosed with HIV and duration of ART were independently associated with disclosure of HIV-positive status to infected children.

Caregivers who had social support were about three times more likely to disclose HIV-positive status to the infected children than those caregivers without social support [AOR = 2.7, 95% CI: (1.1–6.4)]. HIV-positive status disclosure was 6.5 times higher in those caregivers who had HIV-positive children in the age group of 10–14 years than those caregivers who had HIV-positive children less than 10 years of age [AOR = 6.5, 95% CI: (2.1–20.2)]. Children diagnosed with HIV in age of 6–11 years had about three times more disclosure than children diagnosed at age less than six years [AOR = 2.8, 95% CI: (1.1–7.1)]. Duration of ART was also a factor that affects the disclosure of HIV-positive status to infected children. Caregivers who have HIV-positive children > 5 years on ART follow-up had about 5 times more chance of disclosure than those who have ART follow-up for five and less years [AOR = 4.7, 95% CI: (1.8–11.2)] (Table 3).

**Table 3:** Associated factors of disclosure of HIV-positive status of school aged children in Bale, Southeast Ethiopia

Variables	Categories	Disclosed *n* (%)	Non-disclosed *n* (%)	COR (95% CI)	AOR (95% CI)	*p*-Value
Sex of caregivers	Male	25 (43.9)	41 (28.7)	Ref.	Ref.	
	Female	32 (56.1)	102 (71.3)	.73 (0.3, 1.9)	–	
With whom children is living	Biological parent	35 (61.4)	107 (74.8)	Ref.	Ref.	
	None biological parent	22 (38.6)	36 (25.2)	0.10 (0.01, 1.0)	–	
HIV status of care giver	Positive	34 (59.6)	102 (71.3)	Ref.	Ref.	
	Negative	23 (40.4)	41 (28.7)	0.6 (0.3, 1.2)	–	
Residence	Urban	44 (77.2)	92 (64.3)	Ref.	Ref.	
	Rural	13 (22.8)	51 (35.6)	0.7 (0.3, 1.6)	–	
Presence of social support	Present	19 (33.3)	29 (20.3)	Ref.	Ref.	
	Absent	38 (66.7)	114 (79.7)		2.7 (1.1, 6.4)	0.028^a^
Age of children	6–9	5 (8.7)	86 (60.1)	Ref.	Ref.	
	10–14	52 (91.2)	57 (39.9)		6.5 (2.1, 20.2)	0.001^a^
Schooling status of children	Enrolled	55 (96.5)	109 (76.2)	Ref.	Ref.	
	Non-enrolled	2 (3.5)	34 (23.8)	3.2 (1.3, 7.7)	–	
Age at which children were diagnosed with HIV	≤5 year	21 (36.8)	92 (64.3)	Ref.	Ref.	
	6–11	36 (63.2)	51 (35.6)		2.8 (1.1, 7.1)	0.027^a^
Duration of ART	≤5 year	22	114	Ref.	Ref.	
	6–12	35	29		4.7 (1.8, 11.2)	0.001^a^

*Ref* Reference, *AOR* Adjusted Odd Ratio, *COR* Crude Odd Ratio, ^a^ independently associated with disclosure of HIV positive status of children

## Discussion

In this study, only about a quarter of the HIV-infected children were allowed to know their diagnosis result. Disclosing HIV status to children has a number of benefits for children, however, due to different factors, caregivers abstain from revealing their status. This finding is in line with studies done in 2013 and 2014 in northern Ethiopia which showed a prevalence of 31.5 and 33.3%, respectively [15, 19]. In contrary to our study finding, low prevalence of disclosure was reported from Kenya [9, 11], and Addis Ababa (17%) in 2012 [13]. The probable reason for the better prevalence (28.5%) of disclosure in our study could be due to a higher number of older school aged children in the study (mean age of 9.9 years). In addition, the study conducted in Addis Ababa included all pediatric patients whereas our study only focused on school aged children. The time difference between the studies could be another possible reason for observed difference. As the time passes, an awareness is created and different stakehoders work on areas to improve the disclosure status. Still the disclosure in the study area was unacceptably low.

Age of the child was found to be one of the factors independently associated with HIV-positive status disclosure to infected children. Those children in the age group 10–14 years were about seven times more likely to be disclosed of their HIV-positive status as compared to those children whose age was from 6 to 9 years. This could be due to the older children repeatedly asking the reason why they were taking medication. Furthermore, a majority of older children were also in school, and they may have had a chance to learn about HIV. This finding was in agreement with studies conducted in central and northwest Ethiopia where children of the same age were more likely to be disclosed than their counterparts not in school [13, 15]. Similar findings in other African countries reported that children were more likely to know their HIV diagnosis result when older [9, 17]. This result was also supported by qualitative findings of this study.

In this study, more than half of children in the age group 10–14 years were still not disclosed their HIV-positive status. This implies that the majority of HIV-positive children entering secondary sexual characteristics were still unaware of their HIV status. This may result in unknowingly transmitting the virus to others and challenging the strategy of reducing the new HIV infection to zero. This idea was also supported by one of the health care worker participated in in-depth interview. In the HCW in-depth interview, she maintioned that a 14-year-old female infected her boyfriend with the virus unknowingly due to lack of early discloser.

Duration of stay on ART was another factor associated with disclosure of HIV status to children. Those children who were on ART from 6 to 12 years were about five times more likely to be disclosed their HIV status than their counterparts. This finding was in agreement with studies done in developing countries including Ethiopia [19, 20]. The study in Bahirdar reported that children who have taken ART for more than five years were 5 times more likely to be disclosed their status [15]. When the children stay on ART for a long period of time, they might have a chance to ask questions about their HIV medications. The children might ask why they take the medication while they apparently seem healthy. This is also the issue discussed in in-depth interviews.

The age at which children were diagnosed with HIV infection was also associated with disclosure of HIV infection. Childeren who diagnosed with HIV at age above 5 years were about three times more likely to be disclosed their status than those diagnosed at age 5 and less. This may be due to older children asking their caregiver about their diagnosis result. This finding was also supported by qualitative study conducted on the HCWs and caregivers in which they belived that disclosure of HIV status needs maturity of children. Those caregivers who got social support from any source were more likely to disclose the HIV status to infected children than those who didn’t have social support. This may be due to encouragement and social acceptance of the children’s positive status [21].

### Limitations of the study

Clinical characteristics of both children and caregivers were not assessed. Secondary data was used for the children’s information. There may be social desirability bias, and recall bias that might have affected this study. The cross-sectional nature of the study may also have its own limitations. To mimimize this, secondary data was used to cross check some information.

## Conclusions

The prevalence of HIV-positive status disclosure to infected children in Bale zone of Ethiopia was low. Having social support, caring for an older age HIV-positive child, ART follow up for long duration, and HIV diagnosis after age of five years were positively associated with the disclosure of HIV-positive status to infected children. The main reasons for non disclosure were fear of negative consequences for the child, children were too young to understand about HIV, and fear of stigma and discrimination. Health care workers should give age-appropriate counseling, support, and work together with caregivers on the processes of disclosing their diagnostic result to infected children. Non-governmental organizations should strengthen and continue their support for the caregivers on the care of children. Further research should be conducted to address the relationship between disclosure to HIV-infected children and variables like depression, stigma, and adherence involving both children and caregivers.

## Additional file

Additional file 1: Questionnaire used to collect the data in the study (DOCX 26 kb)

## Abbreviations

AIDS: Acquired Immune Deficiency Syndrome
ART: Anti-Retroviral Therapy
HAART: Highly Active Anti-Retroviral Therapy
HCW: Health Care Workers
HIV: Human Immunodeficiency Virus
