# Supplementary material for: Human immunodeficiency virus infection disclosure status to infected school aged children and associated factors in bale zone, Southeast Ethiopia: cross sectional study
Source: BMC Pediatr. 2018 Nov 15;18:356. doi: 10.1186/s12887-018-1336-z (PMC6236985; doi:10.1186/s12887-018-1336-z)
Supplement: Supplementary file 1 — Questionnaire used to collect the data in the study (DOCX 26 kb) [file 12887_2018_1336_MOESM1_ESM.docx]

**Additional file 1**

**Questionnaire**

**Questionnaire to assess the prevalence of HIVpositive disclosure status to infected children and its associated factors in Bale Zone, South East Ethiopia**

**Section One: Socio-demographic characteristics of caregivers**

| **S. No** | **Question** | **Response** | | | **Skip** |
| --- | --- | --- | --- | --- | --- |
| **101** | Age of caregiver | **[______]**in years | | |  |
| **102** | Sex of caregiver | 1. Male 2. Female | | |  |
| **103** | Religion | 1. Muslim 2. Orthodox | 1. Protestant 2. Others specify_______ | |  |
| **104** | Marital status | 1. Single 2. Married | 1. Divorced 2. Widowed | |  |
| **105** | Ethnicity | 1. Oromo 2. Amhara | 1. Tigre 2. Others specify______ | |  |
| **106** | Occupation | 1. Farmer 2. Employer | 1. Merchant 2. Housewife 3. Others specify______ | |  |
| **107** | Educational status | 1. Unable to read andwrite 2. Able to read and write 3. Primary(1-8) | 1. Secondary (9-12) 2. Tertiary (diploma and above) | |  |
| **108** | Monthly income | ____________ (in **ETB**) | | |  |
| **109** | Residence | 1. Urban | | 1. Rural |  |
| **200** | What is your relation to the child | 1. Parents(biological) 2. Grandparent | | 1. Other family member 2. Others specify____ |  |
| **201** | Family size | ________(in number) | | |  |
| **202** | What is your HIV status | 1.Positive | | 2. Negative  3. unknown/not tested | **205**  **205** |
| **203** | Have you disclosed your HIV positive status to anybody | 1. Yes | | 1. No |  |
| **204** | What is your ART status | 1. Before ART 2. On ART | |  |  |
| **205** | Do you have social support | 1. Yes | | 1. No |  |
| **206** | If yes to **Q 205**from whom do you get it? | 1. Community | | 2. Friends  3. NGOs  4.Others specify__________ |  |
| **207** | Do you have support from health care providers on disclosure? | 1. yes | | 1. no |  |

**Section 2 Characteristics of the child on HAART**

| **S.no** | **Question** | **Response** | | **skip** |
| --- | --- | --- | --- | --- |
| 300 | Age of the child? | _________in years | |  |
| 301 | Sex of the child? | 1. Boy 2. Girl | |  |
| 302 | Age of the child at the diagnosis of HIV | _________in years | |  |
| 303 | Age of the child when ART was initiated | ________in months/years | |  |
| 304 | Duration on ART | __________in months | |  |
| 305 | **Is the child responsible to take his/her drug?** | 1. Yes 2.No | |  |
| 306 | Educational status of the child | 1. Not started education 2. Kindergarten(KG) 2. Primary school 4. other specify_________ | |  |
| 307 | With whom the child is currently living | 1. Biological parent 2. Grandparents 3. Relatives | 1. At orphanage camp 2. Others specify_________ |  |
| 308 | Did the child lose any of his/her biological parents? | 1. Yes | 1. Nogo to **Sec 3** | es |
| 309 | If yes to **Q 308** whom did he/she lose? | 1. Mother only 2. Father only | 1. Both 2. I don’t know |  |

**Section 3: Disclosure of HIV status of the child**

| S.no | Question | Response | skip |
| --- | --- | --- | --- |
| 400 | Does the child knew his /her HIV positive status | 1. Yes 2. No | **404** |
| 401 | If **yes** to **Q 500**, who disclosed to the child? | 1. Biological parents 2. Health care providers 3. Grandparents 4. Others specify________ |  |
| **402** | What is/are the reason (s) to disclose to the child?  **Multiple responses are possible** | 1. Childis mature enough 2. Repeated questioning of ‘what happened to me’ by the child 3. Hear from friends or neighbors 4. Child refused to take medications 5. Others specify____________ |  |
| **403** | Age of the child at disclosure | ______________in years |  |
| **404** | Who do you think should be the best person responsible for disclosure of HIV status to the child? | 1. Parents(father or mother) 2. Health care providers 3. Others specify _________ 4. I don’t know |  |
| **405** | What is the appropriate age to disclose HIV positive status to the child | ___________in years |  |

**Section 4: Reason for non-disclosure and intention to disclose (only for non-disclosed)**

| **S.no** | **Questions** | **Response** | **Skip** |
| --- | --- | --- | --- |
| **500** | What is/are the reason(s) for not disclosing HIV positive status to the child? | 1. Fear of socially rejection (fear of stigma and discrimination) 2. Fear of negative consequences for the child (fear of emotional distress) 3. Child is too young to understand the HIV diagnosis. 4. Feel guilty and shame 5. Lack of knowledge and skill 6. Others specify_____________ |  |
| **501** | Do you intend to disclose in the future | 1. Yes 2. No |  |
| **502** | What is the most appropriate time (age) you intend to disclose the HIV diagnosis to your children? | **_______________**in years |  |

**Section five: Checklist to assess clinical characteristics of the children on HAART**

| No | Question | Response | | sss |
| --- | --- | --- | --- | --- |
| 600 | What is the current CD4 count of the child | ______________ | |  |
| 601 | What is the current WHO clinical staging of the child | I 2. II 3. III 4. IV | |  |
| 602 | Does the child have history of hospitalization? | 1. Yes | 1. No 2. I don’t know |  |
| 603 | Does the child have history of opportunistic infections | 1. Yes | 1. No 2. I don’t know |  |

**Thank you**

**Qualitative part**

**IDIs questionnaire for caregivers**

| **S.no** | **Age** | **Sex** | **Relationship to the child** | **Disclosure status of the child** | **HIV status of the caregiver** |
| --- | --- | --- | --- | --- | --- |
|  |  |  |  |  |  |
|  |  |  |  |  |  |
|  |  |  |  |  |  |
|  |  |  |  |  |  |
|  |  |  |  |  |  |
|  |  |  |  |  |  |
|  |  |  |  |  |  |

**Section one A: For disclosed caregivers**

1. What is your opinion about HIV positive status disclosure to HIV infected children?
2. What is your possible reason for disclosing the HIV diagnosis to the child?

Probe for age of the child, maturity of the child, repeated questions from the child, right of the child….

1. What type of information you told the child about their condition (disclosed)?

Probe for the name of the virus, the mode of transmission…

1. What is your experience on HIV Positive status disclosure?

Probe for the outcome of disclosure to the caregiver, to the child

1. What are the benefits of disclosure?

Probe for children’s behavior, ART Adherence

**Section one B: For non-disclosed caregivers**

1. What is your opinion about HIV positive status disclosure to HIV infected children?

Probe for importance, intention to disclose in the future,

1. What is your possible reason for not disclosing the HIV diagnosis to the child?

Probe for fear of social rejection, fear of emotional distress, fear of guilty of transmitting the virus, lack of knowledge and skill

1. What type of information you told the child about their condition?
2. What is your experience on HIV Positive status non-disclosure
3. What are the benefits of disclosure?

Probe for children’s behavior, ART Adherance….

**Section two: IDIs for health care workers**

**Demographic information of health care providers**

| **S.no** | **HI** | **sex** | **Age** | **Educational Background** | **Academic Rank** | **Experience in ART clinic** | **Experience of disclosure to child** |
| --- | --- | --- | --- | --- | --- | --- | --- |
|  |  |  |  |  |  |  |  |
|  |  |  |  |  |  |  |  |
|  |  |  |  |  |  |  |  |
|  |  |  |  |  |  |  |  |
|  |  |  |  |  |  |  |  |
|  |  |  |  |  |  |  |  |

1. Do you think that HIV-infected children should be told about their HIV status?
2. Why it is important to tell children about their HIV status? Probe for the benefits of disclosure,
3. What is the appropriate age to disclose?

Probe for age, appropriate time to disclose

1. Do you have ever disclosed the HIV status to the child?
2. What were the challenges you faced in disclosing HIV status to the child
3. Who do you think should disclose the HIV status to the child?

Probe for HCWs, caregivers or shared? Why?

1. What is your role in disclosing the HIV status to the child? Probe for support, provide HE, provide ongoing counseling,
2. What kind of support do you need to facilitate disclosure to HIV infected children?

Probe for workshop and training, guideline
